# The Antitumor Effect of Curcumin in Urothelial Cancer Cells Is Enhanced by Light Exposure In Vitro

**DOI:** 10.1155/2019/6374940

**Published:** 2019-03-11

**Authors:** Frederik Roos, Katherina Binder, Jochen Rutz, Sebastian Maxeiner, August Bernd, Stefan Kippenberger, Nadja Zöller, Felix K.-H. Chun, Eva Juengel, Roman A. Blaheta

**Affiliations:** ^1^Department of Urology, Goethe-University, Frankfurt am Main, Germany; ^2^Department of Dermatology, Venereology, and Allergology, Goethe-University, Frankfurt am Main, Germany

## Abstract

The natural compound curcumin exerts antitumor properties in vitro, but its clinical application is limited due to low bioavailability. Light exposure in skin and skin cancer cells has been shown to improve curcumin bioavailability; thus, the object of this investigation was to determine whether light exposure might also enhance curcumin efficacy in bladder cancer cell lines. RT112, UMUC3, and TCCSUP cells were preincubated with low curcumin concentrations (0.1-0.4 *μ*g/ml) and then exposed to 1.65 J/cm^2^ visible light for 5 min. Cell growth, cell proliferation, apoptosis, cell cycle progression, and cell cycle regulating proteins along with acetylation of histone H3 and H4 were investigated. Though curcumin alone did not alter cell proliferation or apoptosis, tumor cell growth and proliferation were strongly blocked when curcumin was combined with visible light. Curcumin-light caused the bladder cancer cells to become arrested in different cell phases: G0/G1 for RT112, G2/M for TCCSUP, and G2/M- and S-phase for UMUC3. Proteins of the Cdk-cyclin axis were diminished in RT112 after application of 0.1 and 0.4 *μ*g/ml curcumin. Cell cycling proteins were upregulated in TCCSUP and UMUC3 in the presence of 0.1 *μ*g/ml curcumin-light but were partially downregulated with 0.4 *μ*g/ml curcumin. 0.4 *μ*g/ml (but not 0.1 *μ*g/ml) curcumin-light also evoked late apoptosis in TCCSUP and UMUC3 cells. H3 and H4 acetylation was found in UMUC3 cells treated with 0.4 *μ*g/ml curcumin alone or with 0.1 *μ*g/ml curcumin-light, pointing to an epigenetic mechanism. Light exposure enhanced the antitumor potential of curcumin on bladder cancer cells but by different molecular action modes in the different cell lines. Further studies are necessary to evaluate whether intravesical curcumin application, combined with visible light, might become an innovative tool in combating bladder cancer.

## 1. Introduction

Bladder cancer is the second most common urological cancer in Europe and North America [1], whereby transitional cell carcinoma (TCC) accounts for >90% of these patients [2]. The majority of TCC (>70%) is a nonmuscle-invasive bladder cancer (NMIBC), confined to the bladder mucosa and lamina propia, staged as pTa or pT1 tumors. Standard treatment for NMIBC is transurethral resection (TUR), but the risk of recurrence (60–70%) due to reattachment of released tumor cells is high [3]. To prevent tumor recurrence following TUR, intravesical instillation therapy with chemotherapeutic drugs such as mitomycin has been employed [4, 5]. Due to a higher efficacy against pT1 and pTcis TCC, intravesical bacillus Calmette-Guerin (BCG) immunotherapy has become the treatment of choice over the past three decades. However, cystitis and systemic toxicity are serious side effects [2]. Furthermore, a number of patients suffering from TCC are resistant to conventional intravesical therapy, necessitating the development of less toxic, but effective drugs to combat the disease.

The use of nutraceuticals and functional foods as an alternative or complementary (CAM) option for the prevention and treatment of human disease has gained high popularity. Indeed, natural products play an indispensable role as drugs, supplements, and lead compounds for patients suffering from cancer. About 50% of patients applying CAM-methods employ natural herbs [6–8], either to boost the immune system, to actively contribute to the tumor therapy, and/or to lower the risk of cancer relapse [9].

Still, though natural plant products are well accepted and their application rates high, scientifically based information about their antitumor efficacy is sparse and the herbal compound curcumin is no exception. Curcumin is a polyphenolic substance extracted from the rhizome of* Curcuma longa*, commonly known as turmeric [10]. Several studies have confirmed that curcumin induces various pharmacological-biological processes, including cell cycle arrest and modulation of cell cycle regulating proteins, along with the induction of apoptosis and suppression of tumor promoting transcription factors [11]. Curcumin also blocks crucial steps of metastasis by altering adhesion molecules involved in metastatic tumor progression [12]. Animal and human studies have shown that curcumin is safe and well tolerated, even when highly dosed [13–16].

Conveying curcumin from the bench to the bedside is challenging, since the compound is poorly absorbed and improperly metabolized, resulting in poor bioavailability. Hence, strategies must be developed to enhance the bioavailability of curcumin and open the way to clinical application. Visible and UVA light have recently been shown to amplify the growth blocking potential of curcumin in cell culture and animal studies [17, 18] and curcumin has proven highly effective in treating psoriatic patients, when activated with light phototherapy [19]. Little is known about the antitumor potential of curcumin in TCC and light activation could exert an effect here as well. Therefore, the effect of curcumin on growth and proliferation of different urothelial cancer cells was investigated to elucidate underlying signal transduction pathways.

## 2. Materials and Methods

### 2.1. Cell Culture

RT112, UMUC3 (ATCC/LGC Promochem GmbH, Wesel, Germany) and TCCSUP (DSMZ, Braunschweig, Germany) bladder carcinoma cells were grown and cultured in RPMI 1640 supplemented with 10% fetal calf serum (FCS), 20 mmol HEPES buffer, 1% glutamax and 1% penicillin/streptomycin (all: Gibco/Invitrogen; Karlsruhe, Germany) in a humidified, 5% CO2 incubator. RT112 is an invasive (pathological stage T2) moderately differentiated (grade 2/3) model of human bladder cancer, UMUC3 a high grade 3 invasive bladder cancer. TCCSUP represents a transitional cell carcinoma, grade 4. Subcultures from passages 7–24 were selected for experimental use.

### 2.2. Curcumin and Light Exposure

Curcumin (Sigma Aldrich, Taufkirchen, Germany) was stored at -20°C and was diluted in cell culture medium to either 0.1, 0.2, or 0.4 *μ*g/ml final concentration prior to use. Tumor cells were treated with curcumin for 1 h. Subsequently, RPMI based cell medium was replaced by phenol red free phosphate buffered saline (PBS) with Ca^2+^/Mg^2+^ (Gibco/Invitrogen) and subjected to visible light exposure (5500 lx, 1.65 J/cm^2^; Waldmann UV 801AL, Villingen-Schwenningen, Germany) for 5 min. After irradiation PBS was replaced by cell culture medium containing no curcumin. Controls were cell cultures exposed to visible light without curcumin, exposed to curcumin without light exposure, or treated with PBS alone. Tumor cells were then subjected to the assays listed below.

### 2.3. Cell Growth, Proliferation, and Apoptosis

We followed the methods of Juengel et al. [20]. Cell growth was assessed using the 3-(4,5-dimethylthiazol-2-yl)-2,5-diphenyltetrazolium bromide (MTT) dye reduction assay (Roche Diagnostics, Penzberg, Germany). Bladder cancer cells (50 *μ*l, 1 × 10^5^ cells/ml) were seeded onto 96-well tissue culture plates. After 24, 48, and 72 h, 10 *μ*l MTT (0.5 mg/ml) were added for an additional 4 h. Thereafter, cells were lysed in a buffer containing 10% SDS in 0.01 M HCl. The plates were incubated overnight at 37°C, 5% CO_2_. Absorbance at 550 nm was determined for each well using a microplate enzyme-linked immunosorbent assay (ELISA, Tecan Infinite M200, Männedorf, Switzerland) reader. After subtracting background absorbance, results were expressed as mean cell number.

Cell proliferation was measured using a BrdU cell proliferation enzyme-linked immunosorbent assay (ELISA) kit (Calbiochem/Merck Biosciences, Darmstadt, Germany). Tumor cells (50 *μ*l, 1 × 10^5^ cells/ml), seeded onto 96-well plates, were incubated with 20 *μ*l BrdU-labeling solution per well for 8 h, fixed and detected using anti-BrdU mAb according to the manufacturer's instructions. Absorbance was measured at 450 nm using a microplate ELISA reader.

To evaluate whether tumor cell growth was impaired or reduced due to apoptosis, the expression of Annexin V/propidium iodide (PI) was evaluated using the Annexin V-FITC Apoptosis Detection kit (BD Pharmingen, Heidelberg, Germany). Tumor cells were washed twice with PBS and then incubated with 5 *μ*l of Annexin V-FITC and 5 *μ*l of PI in the dark for 15 min at RT. Cells were analyzed by flow cytometry using FACScalibur (BD Biosciences, Heidelberg, Germany). The percentage of apoptotic (early and late), necrotic, and vital cells in each quadrant was calculated using CellQuest software (BD Biosciences).

### 2.4. Cell Cycling

Cell cycle analysis was carried out on subconfluent cell cultures. Tumor cell populations were stained with PI, using a Cycle TEST PLUS DNA Reagent Kit (Becton Dickinson, Heidelberg, Germany) and then subjected to flow cytometry using FACScan (Becton Dickinson). 10,000 events were collected from each sample. Data acquisition was carried out using CellQuest software and cell cycle distribution was calculated using the ModFit software (Becton Dickinson). The number of gated cells in the G1, G2/M, or S-phase was expressed as % [20].

### 2.5. Cell Cycle Regulating Proteins

Cell cycle regulating proteins were investigated by Western blot. Tumor cell lysates were applied to a 7%–15% polyacrylamide gel (depending on protein size) and electrophoresed for 90 min at 100 V. The protein was then transferred to nitrocellulose membranes (1 h, 100 V). After blocking with nonfat dry milk for 1 h, the membranes were incubated overnight with monoclonal antibodies directed against the following cell cycle proteins, all from BD Biosciences, Heidelberg, Germany, if not otherwise indicated: Cdk1 (IgG1, clone 1, dilution 1:2,500), phospho-Cdk1 (pTyr15; IgG1, clone 44), cdk2 (IgG2a, clone 55, dilution 1:2,500), phospho-Cdk2 (Thr160; MerckMillipore, Darmstadt, Germany), cyclin A (IgG1, clone 25, dilution 1:250), cyclin B (IgG1, clone 18, dilution 1:1,000), cyclin D1 (IgG1, clone G124-326, dilution 1:250), Skp1 p19 (IgG1, clone 52/p19, dilution 1:5,000), and p27 (IgG1, clone 57, dilution 1:500).

To investigate histone acetylation, cell lysates were marked with antiacetylated histone H3 (rabbit IgG, clone Y28, dilution 1:500, Epitomics, USA) and antiacetylated histone H4 (Lys8, rabbit IgG, dilution 1:500, Upstate Biotechnology, USA).

HRP-conjugated goat anti-mouse IgG (Upstate Biotechnology, Lake Placid, NY, USA; dilution 1:5,000) served as the secondary antibody. The membranes were briefly incubated with ECL detection reagent (ECLTM, Amersham/GE Healthcare, Munich, Germany) to visualize the proteins and then were analyzed by the Fusion FX7 system (Peqlab, Erlangen, Germany). ß-actin (1:1,000; Sigma Aldrich) served as internal control.

Gimp 2.8 software was used to perform pixel density analysis of the protein bands. The ratio of protein intensity/*β*-actin intensity was calculated and expressed in percentage, related to controls set to 100%.

### 2.6. Statistics

All experiments were performed three to six times. Statistical significance was calculated with the Wilcoxon–Mann-Whitney U test. Differences were considered statistically significant at a P value less than .05.

## 3. Results

### 3.1. Effect of Curcumin on Tumor Cell Growth and Proliferation

**Figure 1:**
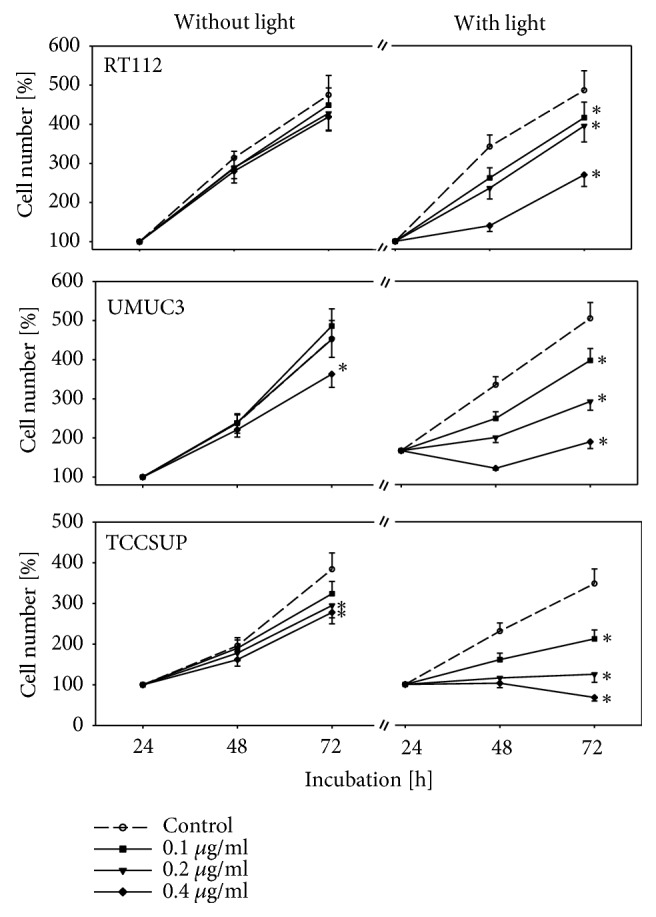
Growth of RT112, UMUC3, and TCCSUP cells treated with curcumin alone or with curcumin plus visible light. Cells were incubated in 96-well-plates for 24, 48, and 72 h. Controls remained untreated. Cell number was set to 100% after 24 h incubation. Bars indicate standard deviation (SD). *∗* indicates significant difference to untreated controls, p ≤ 0.05.

Curcumin alone, added at a concentration from 0.1 to 0.4 *μ*g/ml, did not alter cell growth of RT112 cells, slightly reduced UMUC3 cell growth at 0.4 *μ*g/ml, and only moderately suppressed growth of TCCSUP at 0.2 and 0.4 *μ*g/ml, each compared to respective controls (Figure 1). When RT112 cells were exposed to visible light, curcumin efficacy was considerably enhanced. The same effect was seen with UMUC3 and TCCSUP cells, with a complete growth blockade at 0.4 *μ*g/ml (UMUC3) or already at 0.2 *μ*g/ml (TCCSUP) (Figure 1). Light alone did not influence tumor cell growth (data not shown).

**Figure 2:**
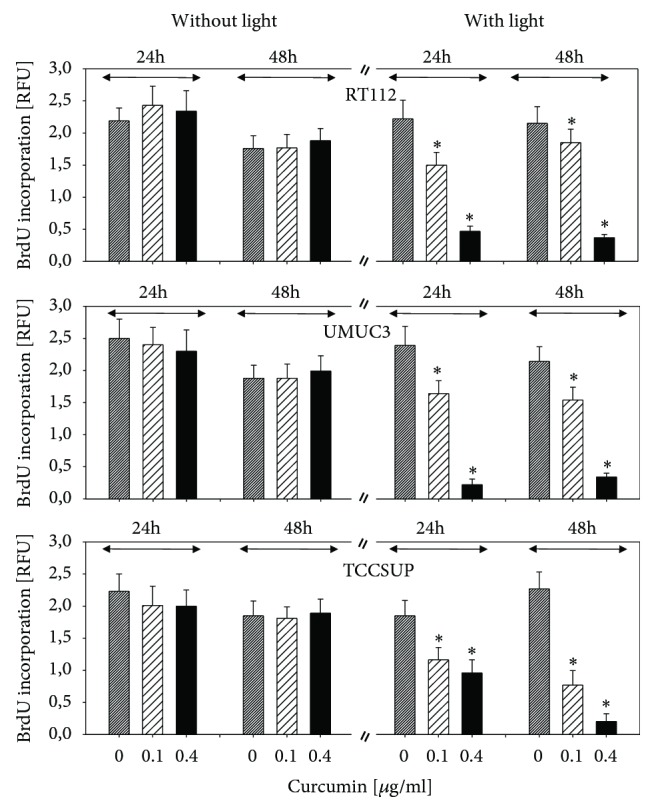
Analysis of tumor cell proliferation. RT112, UMUC3, and TCCSUP cells were exposed to 0.1 or 0.4 *μ*g/ml curcumin alone or with curcumin plus visible light. Controls received cell culture medium alone. BrdU incorporation was evaluated after 24 h or 48 h. The figure depicts relative fluorescence values (RFU). Bars indicate standard deviation (SD). *∗* indicates significant difference to control, p ≤ 0.05.

The BrdU incorporation assay revealed no change in BrdU uptake in cells not treated with visible light, independent of whether tumor cells were evaluated 24 or 48 h after curcumin exposure, independent on drug concentration (Figure 2). Additional light exposure caused BrdU incorporation to become significantly reduced in all three cell lines, with effects already apparent at 0.1 *μ*g/ml (Figure 2). Light alone did not modify BrdU uptake (data not shown).

### 3.2. Effect of Curcumin on Tumor Cell Apoptosis

**Figure 3:**
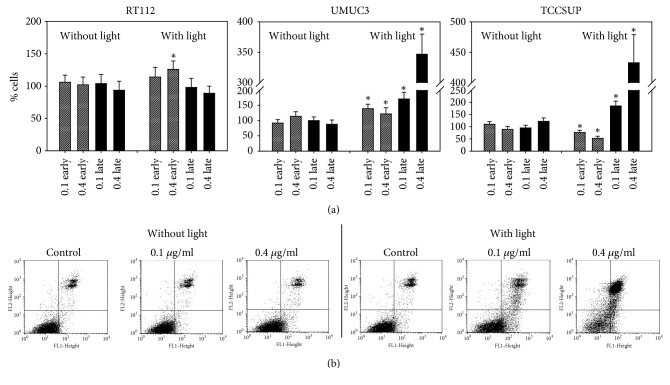
Induction of apoptotic events. RT112, UMUC3, and TCCSUP cells were exposed to 0.1 or 0.4 *μ*g/ml curcumin alone or with curcumin plus visible light. Early and late apoptosis was evaluated as indicated in methods. (a) The number of control cells was set to 100%. *∗* indicates significant difference to control, p ≤ 0.05. (b) Dot blot of one representative test on UMUC3 cells.

Curcumin alone did not trigger apoptotic events in RT112, UMUC3, or TCCSUP cells. Following light exposure 0.4 *μ*g/ml curcumin significantly enhanced the number of early apoptotic RT112 cells, and both early and late apoptotic UMUC3 cells became elevated, with strongest effects evoked at 0.4 *μ*g/ml (Figure 3). A prominent shift towards late apoptosis was observed in TCCSUP cell cultures, particularly with 0.4 *μ*g/ml curcumin.

### 3.3. Curcumin Alters Cell Cycle Progression

**Figure 4:**
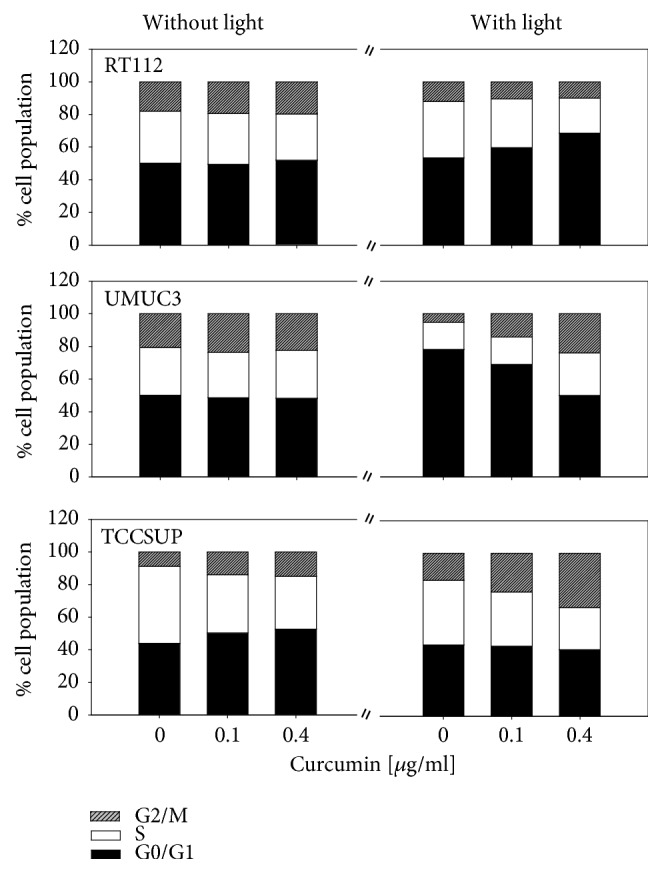
Cell distribution in the different cell cycle phases. Percentage of RT112, UMUC3, or TCCSUP cells in G0/G1, S and G2/M-phase is indicated. Bladder cancer cells were treated with 0.1 or 0.4 *μ*g/ml curcumin alone or with curcumin plus visible light. Controls remained untreated.

Cell cycling of RT112 and UMUC3 cells was not modified when curcumin was added to the cultures without light exposure (Figure 4). The amount of TCCSUP in the G2/M- and G0/G1-phase was upregulated and the number of S-phase cells was downregulated by curcumin when applied at 0.1 and 0.4 *μ*g/ml. Light exposure enhanced the mechanistic activity of curcumin. The number of RT112 cells undergoing G0/G1 phase increased, accompanied by a decrease in the S-phase (Figure 4). The opposite was seen with respect to UMUC3, with a decrease in G0/G1 cells and an increase in G2/M- and S-phase cells. The number of TCCSUP cells in G2/M increased, whereas S-phase cells were diminished. Percentage distribution of cell cycle phases of cell cultures exposed to light, but not treated with curcumin, was similar to the controls (data not shown).

### 3.4. Modification of Cell Cycle Regulating Proteins

**Figure 5:**
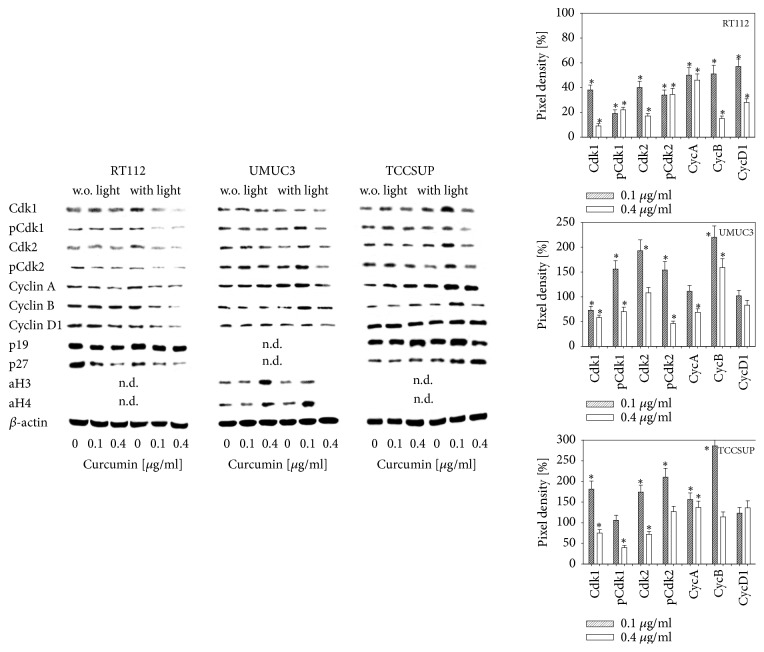
Protein expression profile of cell cycle regulating proteins and histone H3 and H4 acetylation in RT112, UMUC3, or TCCSUP cells after exposure to 0.1 or 0.4 *μ*g/ml curcumin alone or with curcumin plus visible light. Controls (0) received cell culture medium alone. ß-actin served as the internal control. One representative of three separate experiments is shown. w.o. = without; n.d. = not detectable. The right panel shows the pixel density of the curcumin plus light protein bands. Pixel densities for p19, p27, aH3, and aH4 are not shown, since they were not detectable in all cell lines. The ratio of protein intensity/*β*-actin intensity was calculated and expressed as a percentage of the controls, set to 100%. *∗* Significant difference to controls, p≤0.05.

Curcumin did not influence cell cycle protein expression with a few exceptions. The amount of p27 was reduced in RT112 cells at 0.1 and 0.4 *μ*g/ml (0.4 *μ*g/ml > 0.1 *μ*g/ml) curcumin, acetylated H3 and H4 increased in UMUC3 in the presence of 0.4 *μ*g/ml curcumin and the same concentration led to an increase of p19 in TCCSUP (Figure 5). Strong effects were evoked by curcumin after light exposure. Both Cdk1 and 2 (total and phosphorylated), cyclins A, B, and D1 as well as p27 were diminished in RT112 cells. The cell cycle data show that UMUC3 cells behaved differently when curcumin and light were combined. Particularly at 0.1 *μ*g/ml, pCdk1, Cdk2, pCdk2, and cyclin B were elevated, compared to the controls. aH3 was moderately, aH4 was massively upregulated at this concentration. Both histones, however, were not apparent in cells treated with 0.4 *μ*g/ml curcumin. The proteins p19 and p27 were not detectable. Similar to UMUC3, the amount of Cdk-cyclin proteins increased following curcumin treatment and light exposure (0.1 > 0.4 *μ*g/ml). This also became evident with p19 and p27. aH3 and aH4 were not detectable.

## 4. Discussion

Investigations have shown that curcumin potently blocks metastatic tumor progression in vitro [21–23], though chemical instability and poor bioavailability hamper its clinical application. To overcome this limitation, combining curcumin with light has been found to enhance the antitumor activity. Pilot experiments with curcumin alone (without light exposure) revealed that much higher concentrations >5*μ*g/ml were necessary to similarly suppress tumor cell growth as with low-dosed curcumin plus light. This accords with other reports where 5-50 *μ*M curcumin was found necessary to evoke a pharmacological effect [24–26]. The present investigation demonstrates that light exposure combined with low-dosed curcumin (0.1 *μ*g/ml, 0.27 *μ*mol/L) significantly reduces bladder cancer growth and proliferation.

The combination of light with curcumin does not amplify the antigrowth potential of curcumin in bladder cancer cells. Potent antiproliferative effects have also been observed in human keratinocytes with a curcumin concentration of only 0.2 *μ*g/ml, when applied in combination with UVA or visible light [27]. Experimental work on melanoma and oral squamous cell carcinoma cell models have confirmed these findings [18, 28], as has light-induced sensitization of curcumin in a xenograft tumor model with human epithelial carcinoma cells [17]. Thus, the effects of curcumin-light do not seem restricted to a particular type of cancer cell.

Though an inhibitory effect of curcumin-light has been seen with several different cancer types, it is of interest that the molecular mode of action, at least in the investigated bladder cancer cell lines, was different. RT112 cells were arrested in G0/G1, UMUC3 in both the S- and G2/M-phase and TCCSUP in the G2/M-phase, exclusively. These differences were paralleled by differences in cell cycle regulating protein expression. When using 0.1 *μ*g/ml curcumin, the Cdk1-cyclin B and Cdk2-cyclin A axes became deactivated in RT122 cells but were upregulated in UMUC3 and TCCSUP cells. Therefore, the elevation of Cdk1-cyclin B/Cdk2-cyclin A caused by curcumin-light drives UMUC3 and TCCSUP cells into the G2/M and/or the S-phase, whereas downregulation of Cdk1-cyclin B/Cdk2-cyclin A caused G0/G1 phase arrest of RT112 cells. The data obtained with 0.4 *μ*g/ml curcumin deserve particular attention, since Cdks and cyclins were (at least) partially reduced in UMUC3 and TCCSUP cells, as well. A significant increase in the number of apoptotic UMUC3 and TCCSUP cells also occurred. Considering that curcumin induced activation of apoptotic events has been associated with suppression of Cdk1, Cyclin A and B [29, 30], we assume that UMUC3 and TCCSUP cells progress from a G2/M cell cycle arrest to apoptosis by means of a cell signaling switch. The direct transition from G2/M arrest to apoptosis might be uncommon. However, other studies have demonstrated that, prior to apoptosis, tumor cells arrested by curcumin at the G2/M transition point were then released to apoptosis by increasing the curcumin dosage [31]. The present investigation shows that curcumin plus light strongly suppresses growth in all three investigated bladder cancer cells lines, although a congeneric molecular mode of action is not apparent.

How light sensitizes curcumin's effect is not clear, and a primary molecular mechanism has not yet been identified. Barik et al. [32] have observed a high affinity of curcumin to bovine serum albumin, associated with an enhancement of fluorescence emission and a blue-shifting of the fluorescence spectrum. Speculatively, a light dependent energy transfer during curcumin-protein-interaction may enhance the influence of curcumin on protein function and cell regulation [33].

Evidence provided that curcumin in combination with irradiation enhances its antitumor potential on bladder cancer cells. Other antitumor activity such as DNA fragmentation, release of cytochrome c from mitochondria and activation of caspases-9 and -8 caused by the combination of low curcumin concentrations and light have sufficiently been documented for other cancer types [18, 27, 34]. Therefore, curcumin, combined with visible light, seems a reasonable candidate to replace or complement BCG instillation therapy for patients with NMIBC. Indeed, photodynamic therapy through intravesically applied antitumor compounds has already been recommended as a highly effective concept to treat BCG refractory urothelial carcinomas [35, 36]. From a technical viewpoint, Bader et al. presented evidence that transmission of 2 to 6 W white light (100 J/cm2) through a single 1.5mm diameter quartz fiber assembled into a 20 Ch transurethral irrigation catheter might provide optimum activation conditions [37]. Besides treatment of NMIBC, patients not eligible for cystectomy with muscle-invasive bladder cancer (MIBC) may also profit from curcumin and subsequent light activation as an alternative to the trimodal chemotherapeutic treatment regimen formulated by Williams and colleagues [38].

Further studies are now warranted to investigate the effects of curcumin on NMIBC and MIBC in vivo.

## Data Availability

The data used to support the findings of this study are included within the article.

## Abbreviations

TCC: Transitional cell carcinoma
CAM: Complementary and alternative medicine
NMIBC: Nonmuscle-invasive bladder cancer.
